# P-1971. Patterns of T cell receptor usage reveal associations with COVID-19 severity

**DOI:** 10.1093/ofid/ofae631.2129

**Published:** 2025-01-29

**Authors:** Zhongyan Lu, Emily Parsons, Stephanie A Richard, Camille Alba, Gauthaman Sukumar, John Rosenberger, Xijun Zhang, Timothy Burgess, Brian Agan, Clifton Dalgard, Simon Pollett, Allison M Malloy

**Affiliations:** Uniformed Services University of the Health Sciences, Bethesda, Maryland; Uniformed Services University, Bethesda, Maryland; Infectious Disease Clinical Research Program, Department of Preventive Medicine and Biostatistics, Uniformed Services University of the Health Sciences, Bethesda, MD, USA, Bethesda, Maryland; Infectious Diseases, Bethesda, Maryland; Uniformed Services University, Bethesda, Maryland; Uniformed Services University, Bethesda, Maryland; Uniformed Services University, Bethesda, Maryland; Uniformed Services, Bethesda, Maryland; Infectious Disease Clinical Research Program, Department of Preventive Medicine and Biostatistics, Uniformed Services University of the Health Sciences, Bethesda, MD, USA, Bethesda, Maryland; The American Genome Center, Uniformed Services University of the Health Sciences, Bethesda, Maryland; Infectious Disease Clinical Research Program, Department of Preventive Medicine and Biostatistics, Uniformed Services University of the Health Sciences, Bethesda, MD, USA, Bethesda, Maryland; Department of Pediatrics, Uniformed Services University of the Health Sciences, Bethesda, MD, USA, Bethesda, Maryland

## Abstract

**Background:**

T cells are associated with viral clearance and improved outcomes in COVID-19, but also with immunopathology. Defining T cell diversity in response to SARS-CoV-2 infection enables implementation of T cells in prevention and treatment strategies. T cells recognize peptide sequences from pathogens upon presentation in major histocompatibility complexes (MHC). Human MHC molecule diversity results in a wide array of peptide recognition by T cells making viral escape difficult, but also challenging to measure.

**Figure:**
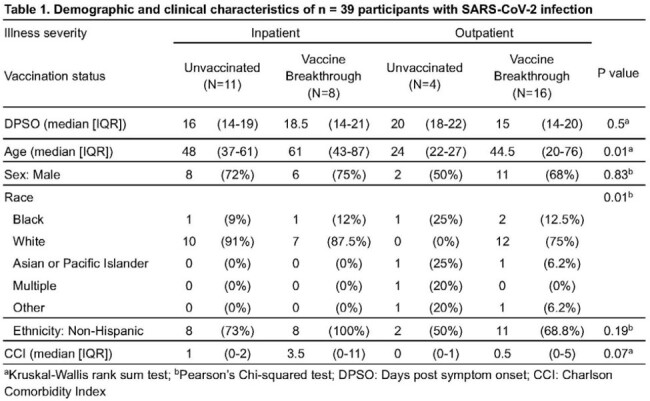

**Methods:**

Using the ImmunoSEQ Assay to sequence complementarity-determining regions in the β chains (CDR3β) of the T cell receptor (TCR) from peripheral blood collected from participants in the EPICC COVID-19 cohort study, we quantified and characterized patterns of TCR usage. Multiple bioinformatic pipelines were compared for analysis.

**Figure:**
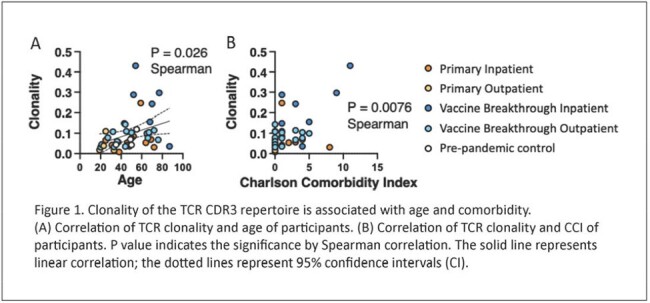

**Results:**

Our study evaluated TCR repertoires from 39 participants who had mild (20 outpatient) versus severe (19 hospitalized) acute COVID-19, and either experienced unvaccinated primary infection (n = 15) or vaccine breakthrough infection (n = 24) (Table 1). From these four clinical groups, we used the ImmuneCODE database, VDJdatabase and healthy controls to identify CD8 TCR sequences and amino acid motifs in the CDR3β region specific for the SARS-CoV-2 proteome. We were surprised to find that vaccine breakthrough and primary infection participants exhibited similar frequencies of SARS-CoV-2-specific TCR sequences over 14-21 days post-symptom onset. Increased use of common TCR clones occurred in participants with older age and comorbidities, which correlated with more severe disease (Figure 1). Strikingly, an increased frequency of non-SARS-CoV-2 TCRs was significantly associated with severe disease in unvaccinated (p = 0.006) and vaccinated participants (p = 0.045).

**Conclusion:**

In conclusion, our study highlights the opportunities and challenges of studying the complexity of T cell diversity and the utility of combining databases and bioinformatics for identification of patterns of TCR recognition. Interestingly, participants who were hospitalized were more likely to have circulating T cells non-specific for SARS-CoV-2 suggesting that vaccines that increase T cell specificity may reduce disease severity.

**Disclosures:**

Simon Pollett, MBBS, AstraZeneca: The IDCRP and HJF were funded to conduct an unrelated phase III COVID-19 monoclonal antibody immunoprophylaxis trial as part of US Govt COVID Response

